# Cytotoxic T cell depletion with increasing epithelial abnormality in women with benign breast disease

**DOI:** 10.1007/s10549-019-05493-5

**Published:** 2020-01-13

**Authors:** Sabina Adhikary, Tanya L. Hoskin, Melody L. Stallings-Mann, Muhammad Arshad, Marlene H. Frost, Stacey J. Winham, Alvaro Peña, Delphine J. Lee, Linda M. Murphy, Michele Rakoff, Lori A. Denison, Keith L. Knutson, Derek C. Radisky, Daniel W. Visscher, Amy C. Degnim

**Affiliations:** 1 Dirks/Dougherty Laboratory for Cancer Research, John Wayne Cancer Institute, Santa Monica, CA USA; 2 Present Address: Kite Pharma Inc., Santa Monica, CA USA; 3 Department of Health Sciences Research, Mayo Clinic, Rochester, MN USA; 4 Department of Cancer Biology, Mayo Clinic, Jacksonville, FL USA; 5 Department of Surgery, Mayo Clinic, 200 First Street SW, Rochester, MN 55905 USA; 6 Department of Medical Oncology, Mayo Clinic, Rochester, MN USA; 7 Department of Medicine, The Lundquist Institute at Harbor-UCLA Medical Center, Torrance, CA USA; 8 Breast Cancer Care and Research Fund, Los Angeles, CA USA; 9 Department of Information Technology, Mayo Clinic, Rochester, MN USA; 10 Department of Immunology, Mayo Clinic, Jacksonville, FL USA; 11 Department of Anatomic Pathology, Mayo Clinic, Rochester, MN USA

**Keywords:** Breast cancer, Cytotoxic T cells, Immunosurveillance, Cancer risk, Benign breast disease, Intraepithelial lymphocytes

## Abstract

**Purpose:**

We quantified cytotoxic T cells in nonmalignant breast tissues from women with and without subsequent breast cancer to assess evidence of whether immunosurveillance may be suppressed prior to tumor development.

**Methods:**

We used an age-matched set of breast tissues from women with benign breast disease (BBD) who subsequently developed breast cancer (BBD with later BC), women with BBD who remained cancer free (BBD cancer-free), and normal Komen Tissue Bank (KTB) tissue donors (KTB controls). We evaluated terminal duct lobular units (lobules) for degree of epithelial abnormality and density of dual-positive CD8/CD103 T cells, as CD103+ cells are thought to be a subset of CD8+ cytotoxic T cells located primarily in the intraepithelial compartment.

**Results:**

In 10 sets of age-matched women, 256 breast lobules were studied: 85 in BBD women with later BC, 85 in BBD cancer-free women, and 86 in KTB donors. The majority of all lobules were histologically normal (*N* = 143, 56%), with 65 (25%) nonproliferative fibrocystic change, and 48 (19%) proliferative epithelial change (with or without atypia). In BBD women with later BC, median CD8+/CD103+ cell density was 39.6, 31.7, and 10.5 cells/mm^2^ (*p* = 0.002) for normal, nonproliferative, and proliferative lobules. In BBD cancer-free women, median CD8+/CD103+ cell density values were 46.7, 14.3, and 0 cells/mm^2^ (*p* = 0.004) respectively. In KTB donors, CD8+/CD103+ cell density was not significantly different across the lobule types (medians 0, 5.8, 10.7, *p* = 0.43).

**Conclusion:**

In women with BBD, breast lobules with increasing epithelial abnormality show significant decreases in cytotoxic T cells as measured by CD8/CD103 staining, suggesting that impaired immunosurveillance may be a component of the earliest stages of breast cancer development.

## Background

The immunosurveillance hypothesis was introduced a century ago by Ehrlich [1] and has gained support with increasing evidence from both animal models and human data that cytotoxic T cells may have a critical role. CD103 expressing CD8 T cells are lymphocytes intimately associated with mucosal surfaces such as intestinal epithelium [2]. They have been called “intraepithelial lymphocytes” (IELs) and have been characterized as a resident memory type of CD8 T cells with potent cytolytic capacities and a role in immune surveillance [3]. Other recent studies show improved outcomes in epithelial tumors with CD103 expressing CD8 T cells such as lung [4], ovarian [5], endometrial [6], and breast cancer [7]. In most of these studies, the CD103-expressing T cells were predominantly intraepithelial and CD103+ cells were more strongly associated with outcomes than CD8+ T cells. We recently reported on immune cells in normal and premalignant breast tissues, with a finding of CD8+ T cells in close association with breast epithelium, representing the main characteristic of mucosal IELs [8]. Therefore, we hypothesized that breast epithelium also harbors similar cytotoxic T cells that may play a role in anti-tumor immunity. Our specific objective for this project was to quantitate dual-positive CD8/CD103 cells in lobules from a case–control set of breast tissues with benign premalignant findings and to investigate associations with breast epithelial abnormalities, as progressive epithelial proliferation in BBD is associated with increasing breast cancer risk [9]. Dual-positive CD8/CD103 stained cells constituted the T cell population of interest as distinct from other CD103 positive cells such as dendritic cells [10].

## Methods

### Study design and breast tissue samples

We obtained Institutional Review Board approval prior to conducting this research. We utilized samples from an existing study of breast tissues from normal donor women and women with benign breast disease [8]. Briefly, normal breast tissues were obtained from the Susan G. Komen^®^ for the Cure Tissue Bank at IU Simon Cancer Center (i.e. Komen Tissue Bank = KTB), a unique resource of tissue from donor women with no known clinical breast abnormalities [11]. Breast tissues with benign disease were obtained from the Mayo benign breast disease (BBD) Cohort [9], a well-annotated cohort of 13,000 women with follow-up information on later breast cancer events. Within the cohort, a nested set of 100 cases (BBD women with later BC) and 100 controls (BBD cancer-free women) were randomly selected from the latter years of the cohort (1992–2001) and were matched on age, year at biopsy, and length of follow-up. Then an age-matched normal breast tissue donor was randomly selected from the KTB samples to create an age-matched triplet: KTB normal tissue donor, BBD woman with later BC, and BBD cancer-free woman. Groups were also frequency matched for first-degree family history of breast cancer. From these age-matched triplets, ten were randomly selected for evaluation of CD8/CD103 staining.

### Histology and immune cell quantitation

For each study sample, an H&E section was digitally scanned with the Aperio ScanScope^®^ XT slide scanner (Leica Biosystems^®^, Buffalo Grove, IL) using the 20 × objective lens. The H&E slide was assessed by the study breast pathologist (DWV) for an overall histologic impression of the greatest severity of abnormality according to established categories of benign breast lesions: no histologic abnormality, nonproliferative changes, proliferative changes without atypia, or atypical hyperplasia. From each digital H&E image, 10 representative lobules (or all lobules if < 10 present) were selected, circled digitally, and annotated using Imagescope. Each circled lobule was individually classified as normal, nonproliferative, or proliferative (with or without atypia), according to established criteria that are based upon the degree of epithelial proliferation and cytologic and architectural atypia, as these categories have stratified later BC risk in multiple studies [9, 12]. Examples of nonproliferative lesions include columnar cell alteration, cystic change, fibroadenoma, apocrine metaplasia; proliferative lesions include columnar cell hyperplasia, usual or florid ductal hyperplasia, radial scar, sclerosing adenosis, flat epithelial atypia, and atypical hyperplasia. A serial formalin-fixed paraffin-embedded tissue section was stained for CD8 and CD103 and also digitally scanned scanned with the Aperio Scanscope^®^ FL slide scanner (Leica Biosystems^®^) using the 20 × objective lens. Matched samples (KTB donor, BBD woman with later BC, BBD cancer-free woman) underwent immunostaining within the same batch to minimize batch effects. The following immunostains were performed with the following antibodies: CD8 (DAKO clone C4/188B, 1:100) and secondary antibody conjugated with AlexaFluor 488 (Life technology, 1:1000); CD103 (AbCam clone EPR4166, 1:2500) and secondary antibody conjugated with AlexaFluor 547 (Life technology, 1:1000).

Samples were deparaffinized with 3 changes of xylene and rehydrated in a series of ethanol baths (100%, 90%, and then 70% Ethanol) and rinsed well in running distilled water. Slides were then placed in a preheated Antigen Retrieval Solution (pH 8.0 EDTA buffer, Vector Lab) for 25 min and then cooled in the buffer for 30 min followed by a 5 min rinse in running distilled water. After the heat-inactivated epitope retrieval step, slides were placed on the DAKO Autostainer for the following procedure (at room temperature). Sections were incubated with phosphate buffered saline (PBS) containing 1% bovine serum albumin (BSA), 2% fetal bovine serum (FBS) to block non-specific staining for 30 min. Sections were incubated in primary antibody at dilutions listed above for 60 min at room temperature. Sections were rinsed with PBS three times, followed by another 60-min incubation with secondary antibody as listed above. The slides were rinsed with PBS three times. Sections were then mounted with 1 drop of Prolong Diamond Antifade Mountant and coverslipped. Digital images were analyzed using Aperio ImageScope Software, version 12.1.0.5029 (Leica Biosystems^®^, Buffalo Grove, IL). The annotated lobules from the H&E slide were identified; then each lobule was circled digitally and the area was calculated. The RGB image was unmixed into separate channels for absorbance at 488 and 594. Within each lobule, positive cells were annotated individually for each color (with all other colors masked). After all positive cells were identified, colors and annotations were unmasked, and both singly and double positive cells were counted.

### Statistical analysis

Cell densities were calculated as the number of positively stained cells per mm^2^ within each outlined lobule area. Number of CD8+/CD103+ cells was analyzed as a count variable using a negative binomial regression model with the logarithm of the lobule area as offset. A generalized linear mixed model approach was used to account for the correlation among multiple lobules measured for each subject. Analysis was performed using PROC GLIMMIX in SAS^®^ (SAS^®^ Institute Inc., Version 9.4). *p* values < 0.05 were considered statistically significant.

## Results

### Characteristics of subjects

The median age was 50 years (range: 40–63 years) and was the same in all three groups because subjects were individually age-matched. Among the 10 BBD cancer-free subjects, median cancer-free follow-up time was 21 years (range 12–27 years). Among the 10 BBD women with later breast cancer, the median time between benign biopsy and cancer development was 8 years (range 2–14 years). The subsequent breast cancer was ER+/PR+ DCIS in one patient and invasive breast cancer in the remaining nine; the invasive breast cancers were 6 HR +/HER2-, 1 HR+/HER2+ , 1 HR-/HER2-, and 1 HR+/HER2 unknown. Four subjects had atypical hyperplasia (a known high risk lesion), two who developed breast cancer and two who remained cancer-free.

### Characteristics of tissue samples and lobules

Among the 30 samples from the 10 age-matched triplets, 256 lobules were evaluated: 86 in normal KTB donors, 85 in BBD women with later BC, and 85 in BBD cancer-free women. The majority of all lobules were histologically normal (*N* = 143, 56%), with 65 (25%) nonproliferative fibrocystic change, and 48 (19%) proliferative epithelial change (with or without atypia), see Table 1. The three groups differed significantly on lobule type distribution (*p* < 0.001), with normal lobules constituting 76% of the lobules in normal KTB donors versus 49% in BBD cancer-free women and 42% in BBD women with later BC. Proliferative lobules were present in 4 normal KTB donors, 4 BBD cancer-free women, and 8 BBD women with later BC.

**Table 1:** Distribution of lobule types in each sample group

	BBD women with later BC *N* = 85 lobules	BBD cancer-free women *N* = 85 lobules	KTB normal donors *N* = 86 lobules	Total *N* = 256 lobules
Lobule type				
Normal	36 (42.4%)	42 (49.4%)	65 (75.6%)	143 (55.9%)
Fibrocystic nonproliferative	21 (24.7%)	32 (37.6%)	12 (14.0%)	65 (25.4%)
Fibrocystic proliferative/atypia	28 (32.9%)	11 (12.9%)	9 (10.5%)	48 (18.8%)

### Distribution and frequency of CD8+/CD103+ T cells

**Fig. 1:**
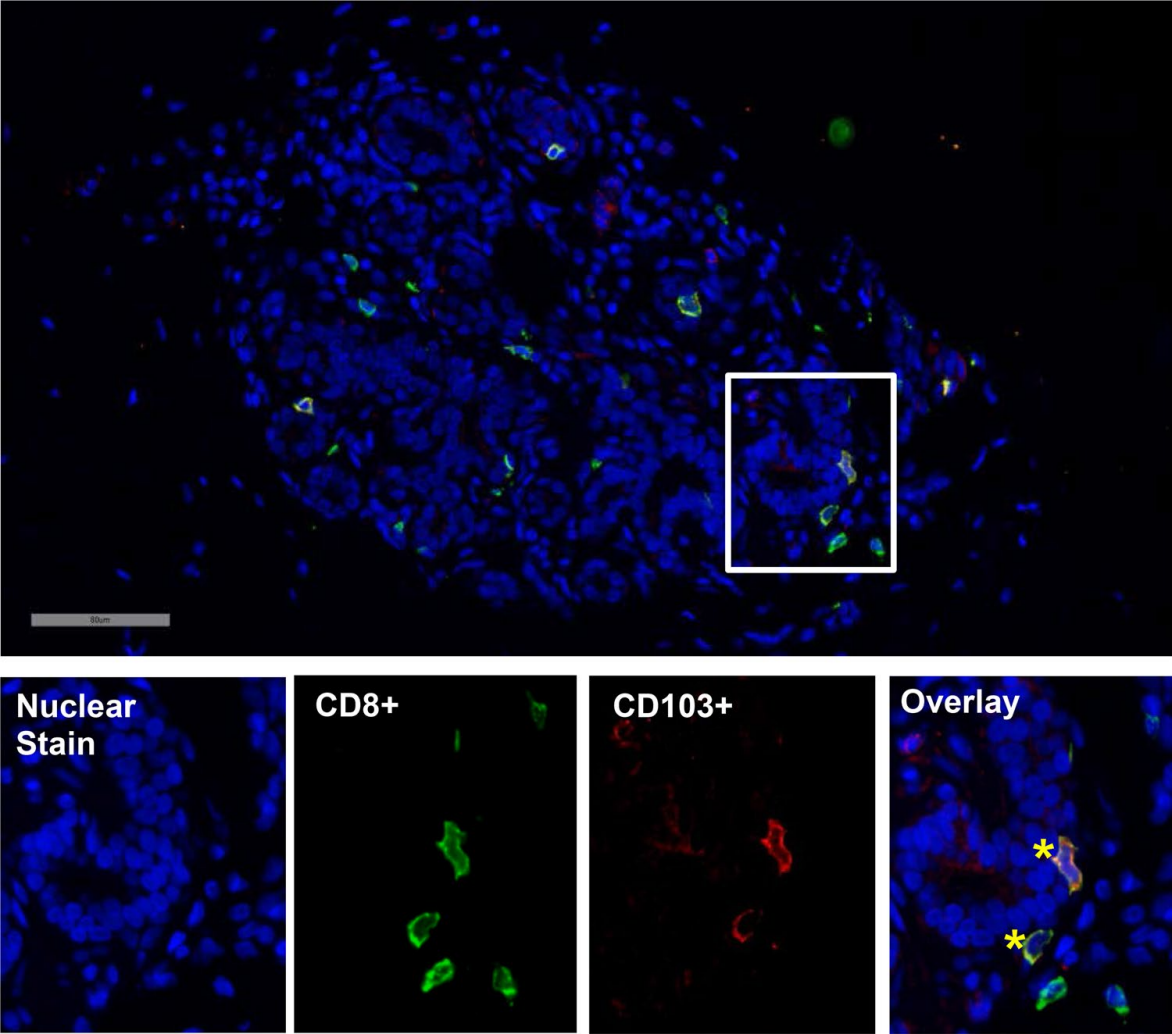
Co-immunofluorescence of CD8 and CD103 in normal mammary lobule. Colocalization of CD8-positive (CD8+) and CD103-positive (CD103+) cells is indicated by asterisks in overlay

**Fig. 2:**
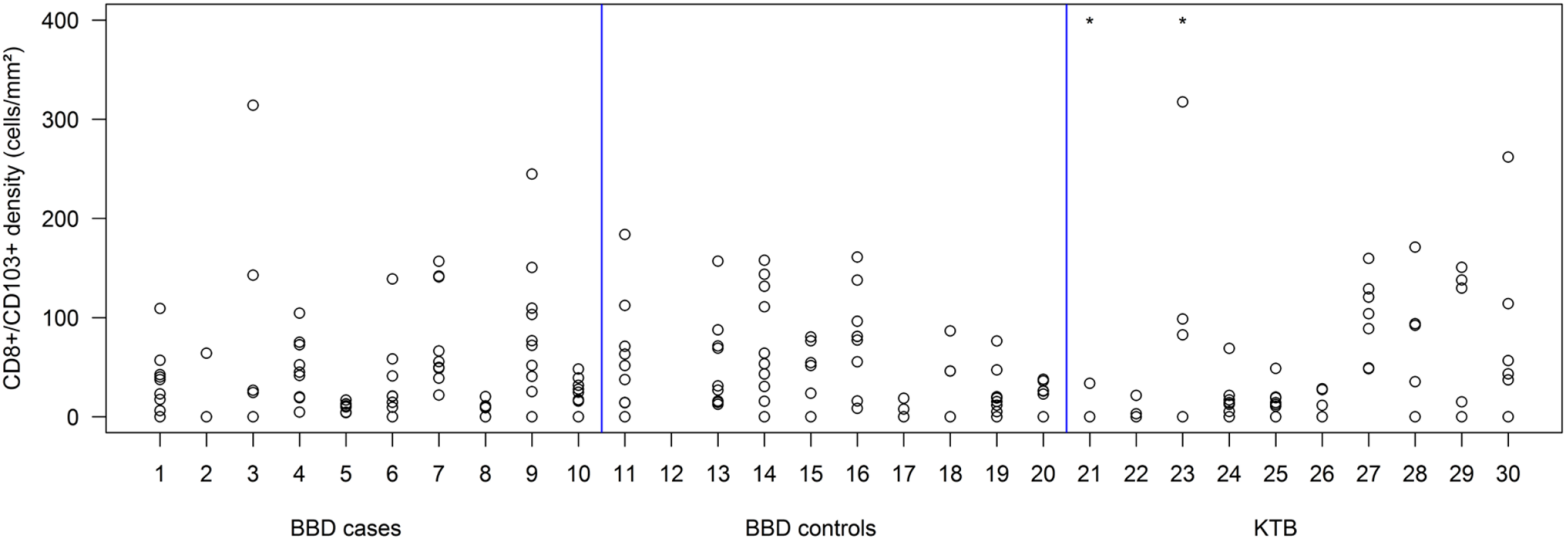
Density of CD8+/CD103+ positive cells per lobule. Ten samples for each category were measured, and up to ten lobules per sample were assessed. Samples with values > 400 cells/mm^2^ are marked with an asterisk. Numbers on the *x*-axis are sample identifiers and do not have any quantitative meaning

T cells stained dual-positive for CD8/CD103 were uniformly located in direct association with epithelial cells (Fig. 1). Density of CD8+/CD103+ cells was right skewed, with 72% of lobules having < 50 cells/mm^2^ and 97% with < 200 cells/mm^2^. CD8+/CD103+ cell density varied both across samples and within the lobules of individual samples (Fig. 2).

### CD8+/CD103+ T cell density and association with epithelial abnormality

**Fig. 3:**
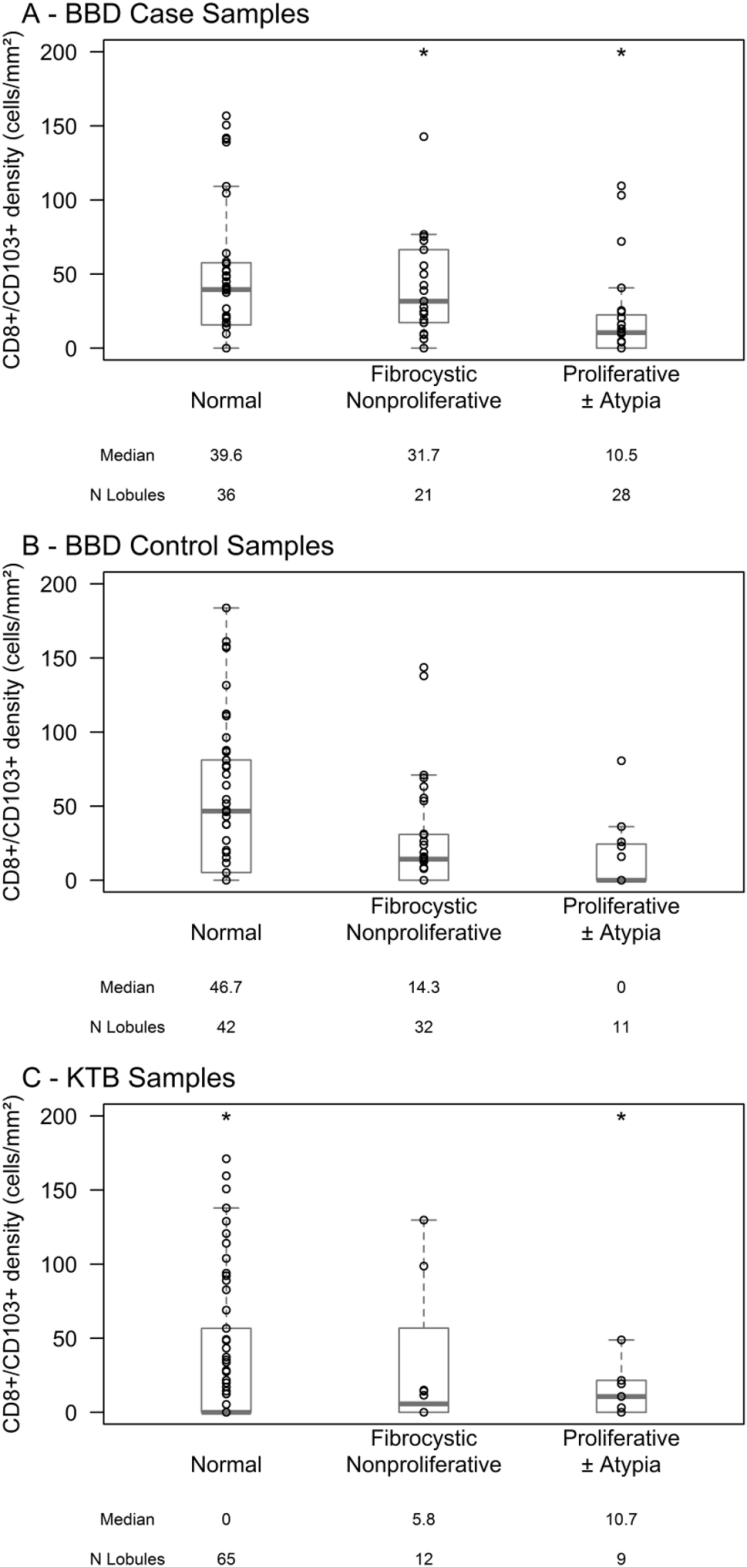
CD8+/CD103+ density by epithelial proliferation. **a** BBD women with later BC (*N* = 85 lobules, *p* = 0.01). **b** BBD cancer-free women (*N* = 85 lobules, *p* < 0.001). **c** KTB normal tissue donors (*N* = 86 lobules, *p* = 0.51). Boxplots demonstrate interquartile range and median values

CD8+/CD103+ cell density was not significantly different across the three sample groups (*p* = 0.98) nor across lobule types in breast tissues from the normal donors (*p* = 0.43, Table 2). However, in benign breast disease tissues, CD8+/CD103+ cell density decreased significantly in lobules with increasing epithelial abnormality, in both BBD women with later BC and BBD cancer-free women (Table 2; Fig. 3). Among BBD women with later BC, median CD8+/CD103+ cell density was 39.6, 31.7, and 10.5 cells/mm^2^ (*p* = 0.002) for normal, nonproliferative, and proliferative lobules. Among BBD cancer-free women, median CD8+/CD103+ cell density values were 46.7, 14.3, and 0 cells/mm^2^ respectively (*p* = 0.004). Only 4 subjects in the study sample had atypical hyperplasia (AH), thus severely limiting our ability to analyze this subgroup separately. Descriptively, however, the same trend observed in the BBD subjects overall (i.e. decreasing CD8/CD103(+) T cell density with increasing lobule abnormality) was present in the patients with AH, with median CD8/CD103(+) T cell density 48.1 cells/mm^2^ in normal lobules, 14.5 cells/mm^2^ in fibrocystic nonproliferative lobules, and 7.9 cells/mm^2^ in proliferative lobules.

**Table 2:** Median (range) of CD8+/CD103+ cell density (#/mm^2^) by lobule type and sample group

	BBD women with later BC	BBD cancer-free women	KTB normal donors
	Median (range) N lobules	Median (range) N lobules	Median (range) N lobules
All lobules	22.0 (0–314.2) *N* = 85	20.2 (0–183.8) *N* = 85	4.2 (0–976.4) *N* = 86
By lobule type			
Normal	39.6 (0–156.9) *N* = 36	46.7 (0–183.8) *N* = 42	0 (0–976.4) *N* = 65
Fibrocystic nonproliferative	31.7 (0–314.2) *N* = 21	14.3 (0–143.7) *N* = 32	5.8 (0–467.4) *N* = 12
Fibrocystic proliferative	10.5 (0–244.7) *N* = 28	0 (0–80.6) *N* = 11	10.7 (0–261.9) *N* = 9

## Discussion

In this study, we found that CD8+/CD103+ lymphocytes are closely associated with the epithelium in breast lobules, and importantly that the density of these cells was significantly lower in lobules with increasing degrees of epithelial abnormality within BBD cases and controls. Although these findings are limited to a small sample size, they are consistent with a protective immunologic function of intraepithelial cytolytic T cells similar to that described in the gut, and they support the possibility of a mucosal immune system in normal human breast that may have a role in tumor immunosurveillance.

CD103 expressing CD8 T cells were first identified as lymphocytes associated with human intestinal epithelium [2] and have been investigated most extensively in that organ. These cells adhere to the epithelial cells via the interaction of CD103 with epithelial E-cadherin [13] and can directly lyse epithelial tumor cells by releasing lytic granules such as granzyme B upon epithelial E-cadherin and CD103 interaction [14, 15], thus helping to maintain epithelial integrity. Lymphoepithelial interactions have been reported to play a major role in mucosal homeostasis in the gut. Secreted growth factors and cytokines as well as the physical interactions between the two cell types help keep the homeostasis. This balance can easily be disrupted during chronic inflammation. In benign breast disease tissues, we observe decreasing CD8+/CD103+ cells with increasing epithelial proliferation. This is particularly interesting given that our previous study showed higher densities of other immune cell types in lobules of BBD compared to normal tissues, suggesting greater inflammation in these tissues [8]. Activation of NFkb, a hallmark of inflammation, is a major factor in downregulation of E-cadherin in epithelial cells [16]. Our observations that CD8+/CD103+ cell density (1) decreased with increasing epithelial proliferation in BBD lobules, and (2) did not differ significantly across lobule types in breast tissues from normal donors are compatible with other published results on IELs and chronic inflammation in the gut mucosa and thus consistent with a possible mechanism of insufficient homing and impaired surveillance.

## Conclusion

In conclusion, in women with BBD, breast lobules with increasing epithelial abnormality show significant decreases in cytotoxic T cells as measured by CD8+/CD103+ staining. These early findings in a small sample size add support to further study the theory that a dysfunctional immune state and impaired tumor immunosurveillance contribute to evolution of breast cancer.

## Data Availability

The datasets used and/or analyzed during the current study are available from the corresponding author on reasonable request, conditional upon approval of the request by the Mayo Clinic Institutional Review Board.

## Abbreviations

BBD: Benign breast disease
KTB: Komen Tissue Bank
IEL: Intraepithelial lymphocyte
